# Spectroscopic detection of traumatic brain injury severity and biochemistry from the retina

**DOI:** 10.1364/BOE.399473

**Published:** 2020-10-08

**Authors:** Carl Banbury, Iain Styles, Neil Eisenstein, Elisa R. Zanier, Gloria Vegliante, Antonio Belli, Ann Logan, Pola Goldberg Oppenheimer

**Affiliations:** 1 School of Chemical Engineering, The University of Birmingham, Edgbaston, Birmingham, B15 2TT, UK; 2 Computer Science, The University of Birmingham, Edgbaston, Birmingham, B15 2TT, UK; 3 Department of Neuroscience, Istituto di Ricerche Farmacologiche Mario Negri IRCCS, Milan, Italy; 4 Institute of Inflammation and Ageing, The University of Birmingham, Edgbaston, Birmingham, B15 2TT, UK

## Abstract

Traumatic brain injury (TBI) is a major burden on healthcare services worldwide, where scientific and clinical innovation is needed to provide better understanding of biochemical damage to improve both pre-hospital assessment and intensive care monitoring. Here, we present an unconventional concept of using Raman spectroscopy to measure the biochemical response to the retina in an *ex-vivo* murine model of TBI. Through comparison to spectra from the brain and retina following injury, we elicit subtle spectral changes through the use of multivariate analysis, linked to a decrease in cardiolipin and indicating metabolic disruption. The ability to classify injury severity via spectra of the retina is demonstrated for severe TBI (82.0 %), moderate TBI (75.1 %) and sham groups (69.4 %). By showing that optical spectroscopy can be used to explore the eye as the window to the brain, we lay the groundwork for further exploitation of Raman spectroscopy for indirect, non-invasive assessment of brain chemistry.

## 1. Introduction

Traumatic brain injury (TBI), resulting from sudden impact such as assault, sporting injuries or road traffic accidents is a major cause of morbidity and mortality, affecting an estimated 69 million individuals worldwide each year [1]. The initial damage triggers a complex cascade of metabolic, biochemical and inflammatory responses leading to secondary injury that can occur over the following hours, days or months [2]. The Glasgow coma scale (GCS), based on visual assessment of the patient’s verbal, visual and motor responses, is the current gold standard to stratify injury severity and acute clinical evolution in TBI. The GCS defines arbitrary boundaries for injury severity grouped as mild, moderate and severe [3]. Whilst this has real clinical value, minimal mechanistic insight is provided into the pathobiology of damage evolution after injury. Novel technologies which can be applied quickly and non-invasively at the point of care (PoC) for interfacing with the brain and define the chemical signatures of TBI pathobiology are needed. A non-invasive method that can detect and quantify TBI would not only provide a more accurate, objective and timely approach to diagnosis, but may help expand our understanding of injury evolution and enable personalized intervention approaches.

The skull provides a thick protective layer around the brain, which strictly limits the available options for both non-invasive and invasive sampling of brain tissue, especially in pre-hospital settings. However, sitting at the back of the eye exists a small part of the brain covered only by optically clear media; the retina and the optic disc. The optic disc appears as a bright circle in fundus images, and is the route through which visual information captured from the retina is passed to the brain along the optic nerve. Also known as the blind spot, the optic disc is devoid of photoreceptive cells and consists predominantly of white matter. Derived from an out-pouching of the diencephalon as the brain develops and bathed in the cerebrospinal fluid, both the optic nerve and retina are technically part of the central nervous system [4]. The retina and optic nerve have long been known to display physically measurable changes as a result of increased intracranial pressure (ICP), where ICP monitoring is of paramount importance for intensive care monitoring in TBI. Measurements from the eye of such changes have been the target of several studies aiming to develop non-invasive ICP, but to our knowledge no such attempts have been made to measure the resultant biochemical change [5–7].

We therefore have hypothesized that a form of optical spectroscopy, which has the potential to be translated into a non-invasive method to probe the posterior segment of the eye (retina and optic nerve), may be able to monitor injury evolution in real time after TBI. Among the optical spectroscopy techniques, Raman spectroscopy offers the richest and most sensitive chemical discrimination. Temporal changes from direct analyses of brain tissue have previously been studied by Surmacki *et al.* [8] using our murine model of TBI. Raman scattering is based on the inelastic interaction between light and a molecule, where the energy exchange from a scattering event causes a change in the vibrational energy level of a molecular bond. Since energy levels of electrons in molecules are quantized, only specific and discrete energy states are allowed. The Raman spectrum therefore defines a chemical fingerprint that is uniquely determined by the underlying molecular constituents [9]. Nevertheless, for biological samples there exists significant redundancy and complexity of spectral bands that makes analysis and interpretation of the data non-trivial. Raman scattering is also an extremely weak effect, and thus, long acquisition times and the use of a high-powered laser focused through an objective are standard requirements for well resolved spectra. The notion of laser exposure to the eye invokes a natural aversion, however every anatomical tissue layer of the eye has been successfully studied using Raman spectroscopy [10]. By adhering to laser safety limits, Obana *et al.* [11] conducted an *in-vivo* study using resonance Raman spectroscopy in humans to assess age-related maculopathy. More recently, Marro *et al.* [12] were able to measure inflammatory changes in retina cell cultures and Stiebing *et al.* [13] have showed how this can be extended to non-resonant Raman spectroscopy, using flat mounted retina samples combined with an optical pathway mimicking the human eye.

Recently, we developed a machine learning technique based on self organizing maps (SOM)s, the self optimizing Kohonen index network (SKiNET) for simultaneously providing rich information and classification from biological samples, even with noisy or poor quality spectra that would result from a lower laser power and short acquisition times [14]. SOMs provide visually intuitive 2D clustering (e.g. according to injury state) of high dimensional data such as Raman spectra, that are otherwise difficult to interpret for large sample and measurement numbers. Whilst SOMs are usually an unsupervised method, SKiNET incorporates supervised learning to additionally provide accurate classification, which could then be used to make diagnostic predictions. Finally, a form of feature extraction using the self organizing map discriminant index (SOMDI) allows us to understand which spectral features (and therefore chemical changes) are responsible for the clustering seen in the SOM [15].

Here, Raman spectroscopy combined with SKiNET is applied to investigate whether the retina can reflect the brain microenvironment after injury, in a clinically relevant murine model of focal TBI. Our results show that spectra from the eye can distinguish moderate TBI (mTBI) and severe TBI (sTBI) from a sham group, and show this to be as a result of similar chemical changes to those seen at the point of injury on the brain. Through quantitative and qualitative analysis we suggest the detected changes are largely due to metabolic distress and the release of cardiolipin, consistent with recent work in the field of mass spectrometry [16]. This validation is particularly promising as mass spectrometry provides vastly superior molecular discrimination. However, Raman spectroscopy has the advantage of being a non-destructive technique and as highlighted, there are ongoing efforts in the field for translation into *in-vivo* measurements, and diagnostics.

## 2. Methods

### 2.1 Mouse model of TBI and tissue processing

Adult (8 weeks old) C57BL/6J male mice (Envigo RMS srl) were used. No additional procedures were performed on mice except those related to the experiment they were intended for. Procedures involving animals and their care were conducted in conformity with the institutional guidelines at the Istituto di Ricerche Farmacologiche Mario Negri IRCCS, Italy in compliance with national (D.lgs 26/2014; Authorization n. 19/2008-A issued March 6, 2008 by Ministry of Health) and international laws and policies (EEC Council Directive 2010/63/UE; the NIH Guide for the Care and Use of Laboratory Animals, 2011 edition). They were reviewed and approved by the Mario Negri Institute Animal Care and Use Committee that includes ad hoc members for ethical issues, and by the Italian Ministry of Health (Decreto no. D/07/2013-B and 301/2017-PR). Animal facilities meet international standards and are regularly checked by a certified veterinarian who is responsible for health monitoring, animal welfare supervision, experimental protocols and review of procedures. Mice were anesthetized by isoflurane inhalation (induction 3%; maintenance 1.5%) in an $N_{2}$O/$O_{2}$ (70%/30%) mixture and placed in a stereotaxic frame. Rectal temperature was maintained at 37 °C. Mice were then subjected to craniectomy followed by induction of controlled cortical impact brain injury as previously described [17]. Briefly, the injury was induced using a 3-mm rigid impactor driven by a pneumatic piston rigidly mounted at an angle of $20^{\circ }$ from the vertical plane and applied to the exposed dura mater, between bregma and lambda, over the left parietotemporal cortex (antero-posteriority: −2.5 mm, laterality: −2.5 mm), at an impactor velocity of 5 m/s. The deformation depth was of either 1 mm or 0.5 mm, resulting in a severe (sTBI) or moderate (mTBI) level of injury respectively. The craniotomy was then covered *via* cranioplasty and the scalp sutured. Sham mice received identical anesthesia and surgery without brain injury. Three days after TBI, mice were deeply anesthetized with Ketamine Chlorhydrate (150 mg/kg, i.p.) and Medetomidine Chlorhydrate (0.2 mg/kg, i.p.) transcardially perfused with 30 mL of phosphate-buffered saline (PBS) 1% (pH 7.4), followed by 60 mL of paraformaldehyde (PFA) 4% in PBS. The brains and eyes were carefully removed from the skull and post-fixed in 4% PFA in PBS for 24 hours at $4^{\circ }$C. The post-fixed tissue were then rinsed and stored in normal saline (NaCl 0.9%) at 4°C. Samples we mounted on microscope slides covered with aluminum foil for spectroscopy studies as whole brains. Retina samples were prepared by micro-dissection of eyes in PBS, followed by flat mounting on aluminum slides. Samples were air dried for 1 hour before measurement.

### 2.2 Raman spectroscopy

An InVia Qontor (Renishaw plc) equipped with a 785 nm laser was used for all measurements. Raman maps over a 20x20 grid (400 spectra), using a step size of 1.5 µm between points were acquired for each sample. The surface map feature in the instrument software (WiRE (Renishaw plc)) was used to follow the topography of the sample, by uniformly measuring 9 position coordinates (x,y,z) and interpolating over the map area. At each position, a spectrum was recorded using a laser power of 50 mW, focused through a 50x Leica objective (0.75 NA) over 5 s (1 s acquisition, 5 accumulations). Spectra were measured in the range 605-1715 ${\mathrm{cm}}^{-1}$ using a 1200 l/mm grating. Instrument calibration was performed using the internal silicon reference built into the InVia system. Care was taken to ensure consistent sample preparation between samples and across injury states. All tissue was kept refrigerated in PBS prior to measurement. All Raman spectra were measured on the same day for each tissue type (brain, retina) and within 72 hours of sacrifice. Raman maps were measured in the contusion core for mTBI and sTBI, and the corresponding area in the sham group. Maps measured from the retina of both eyes were taken from an area in close proximity to the optic disc for each mouse. Post processing of spectra was performed in WiRE 5.3 (Renishaw Plc), cosmic rays were removed from each map using the nearest neighbor method, followed by baseline subtraction using the ‘intelligent spline’ fitting (11 nodes). Finally, the average was taken from each map resulting in a single spectrum per sample.

### 2.3 Analysis of retina tissue

The 400 spectra measured across each tissue sample were grouped according to injury state from both eyes (summarized in Table 1). 20 % of the data was randomly selected from each group and reserved as test data, leaving the remaining 80 % for training (Table 2). Analysis of the training data was performed using SKiNET [18], by randomly passing samples from the training data into the SOM over a number of iterations. SKiNET models were optimized by performing 10 fold cross validation on the the training data, and tuning the number of neurons, initial learning rate and number of training steps. The final model used a 20x20 grid of neurons, 57600 training steps (5 epochs of the data), with an initial learning rate of 0.2. The initial neighborhood size was maintained at 2 / 3 the edge length of the grid and cosine similarity used as the distance metric to determine the best matching unit. Finally, the optimized model was used to classify the previously unused test data, to give an indicator of the classification performance. Classification using the test data were repeated 10 times from separate SOM initializations and an average of the results output as a confusion matrix. An illustration of the workflow is shown in Fig. S1.

**Table 1.** Summary of retina spectra used as inputs for multivariate analysis across the three injury states (sham, mTBI and sTBI).

	Spectra Per Tissue Sample	Mice	Eyes	Total

Sham	400	6	2	4800
mTBI	400	6	2	4800
sTBI	400	6	2	4800

				14,400

**Table 2.** Breakdown of data across each injury state, and split into training and test data sets.

Injury State	Total	Training Data (80 %)	Test Data (20 %)

Sham	4800	3840	960
mTBI	4800	3840	960
sTBI	4800	3840	960

Total	14,400	11,520	2880

### 2.4 Analysis of brain tissue

SkiNET was used to analyze spectra measured from whole brain samples as described in the previous section. The 400 spectra measured across each tissue sample were grouped according to injury state from the contusion core, and 20 % of the data reserved as test data (summarized in Table 3). Non-negative least squares (NNLS) analysis was performed on brain tissue by fitting a library of component spectra to the average spectrum for each brain sample (Fig. S2d-f). The component spectra consisted of raw data provided by Krafft *et al.* [19] for human brain lipids and cardiolipin. Cytochrome c was purchased from Sigma-Aldrich Ltd and measured without modification at 785 nm using a laser power of 10 mW, focused through a 50x Leica objective (0.75 NA) over 10 s (1s acquisition, 10 accumulations). The **lsqnonneg** function in MATLAB was used to determine coefficients of the raw component spectra to the average spectra measured from the brain. The **interp1** function was used to rescale the data in the range of 1200-1714 ${\mathrm{cm}}^{-1}$ in increments of one inverse centimeter.

**Table 3.** Breakdown of data across each injury state, and split into training and test data sets from brain tissue in the contusion core.

Injury State	Total	Training Data (80 %)	Test Data (20 %)

Sham	2400	1920	480
mTBI	2400	1920	480
sTBI	2400	1920	480

Total	7200	5760	1440

## 3. Results

**Fig. 1.**
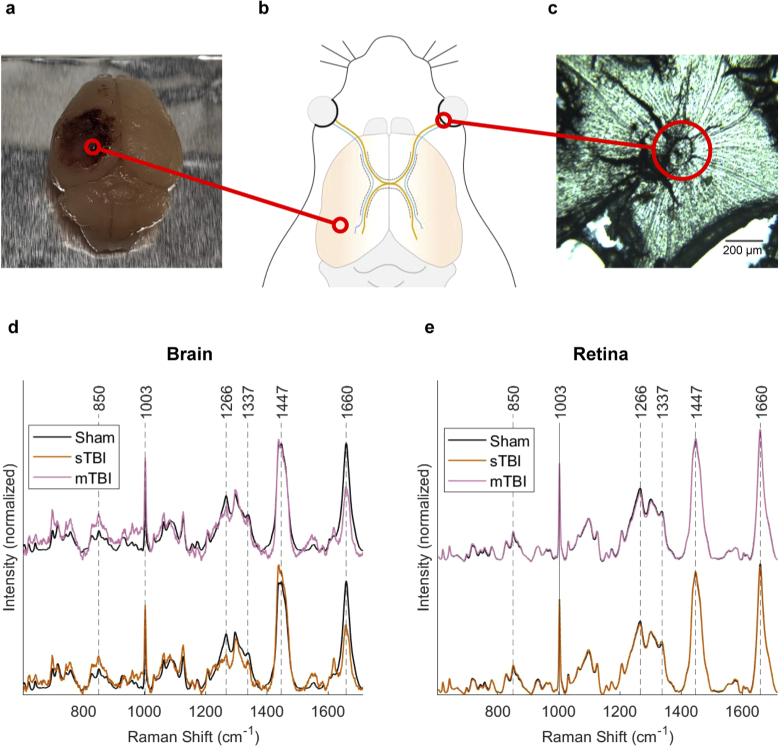
**a**, Photograph of whole brain following sTBI to the left parietotemporal cortex. **b**, Illustration of mouse brain and optic tract, highlighting ipsilateral (blue) and contralateral (orange) projections connecting the brain to the retina. **c**, Example of a bright field microscopy image of a flat mounted retina from the mTBI group. **d**, Average Raman spectra collected in the contusion core for mTBI and sTBI compared to the sham group. **e**, Average Raman spectra collected from flat mounted retina samples (both eyes), showing mTBI and sTBI compared to the sham group. Raman spectra were collected as map measurements of 400 spectra over each sample, using a 785 nm excitation laser (50 mW), 1s acquisitions with 5 accumulations. Map measurements from each sample were averaged to produce a single spectrum per sample (n=6 for each injury state).

Experimental TBI was induced by controlled cortical impact in mice (n=6 for each injury state), with the degree of injury (either moderate or severe) being defined by the deformation depth. Tissue samples of postfixed brain (Fig. 1(a)) and eyes were collected 3 days after injury from sham, mTBI and sTBI groups. An illustration of the mouse head is shown in Fig. 1(b), highlighting the bilateral axon projections that are present between the brain and the retina. Each eye was carefully dissected to isolate and flat mount the retina as shown in Fig. 1(c). The corresponding Raman spectra (averaged over all samples) from the contusion core of the brain (Fig. 1(d)) and from flat mounted retina (Fig. 1(e)) are shown for mTBI and sTBI against the sham group. Assignments to the highlighted bands are summarized in Table 4, along with common biochemical attributions, with reference to the database published by Talari *et al.* [20].

**Table 4.** Summary of chemical assignments and biochemical attribution to Raman bands which display a change after TBI. Assignments were made with reference to Larkin [21], common biochemical attributions made with reference to the database by Talari *et al.* [20].

Peak (${\mathrm{cm}}^{-1}$)	Assignment	Attribution

850	C-H wagging	-
1003	C-C skeletal	phenylalanine
1266	C-H bending	mixed (proteins/lipids)
1337	C-N stretching, N-H bending	Amide III
1447	${\mathrm{C-H}}_{2}$ bending	mixed (proteins/lipids)
1660	C=C stretching	mixed (proteins/lipids)

### 3.1 Multivariate analysis of retina tissue

From the average spectra of brain tissue in Fig. 1(d), dramatic changes to the bands around 1266 and 1660 ${\mathrm{cm}}^{-1}$ are clearly visible, however the data from the retina (Fig. 1(e)) are almost indistinguishable across the three injury states. The average spectra provide an easily digestible format in order to present the data, but forces us to throw away vital information that arises from point-point variation within each sample combined with sample-sample variation. Fortunately, multivariate techniques allow us to capture all of this information and extract the most important spectral features which characterize a group of data, such as an injury state. Recently, we highlighted the value of SOMs in the analysis of Raman spectra from biological samples [14]. The 400 spectra measured across each tissue sample were grouped according to injury state from both eyes. 20 % of the data was randomly selected from each group and reserved as test data, leaving the remaining 80 % for training.

**Fig. 2.**
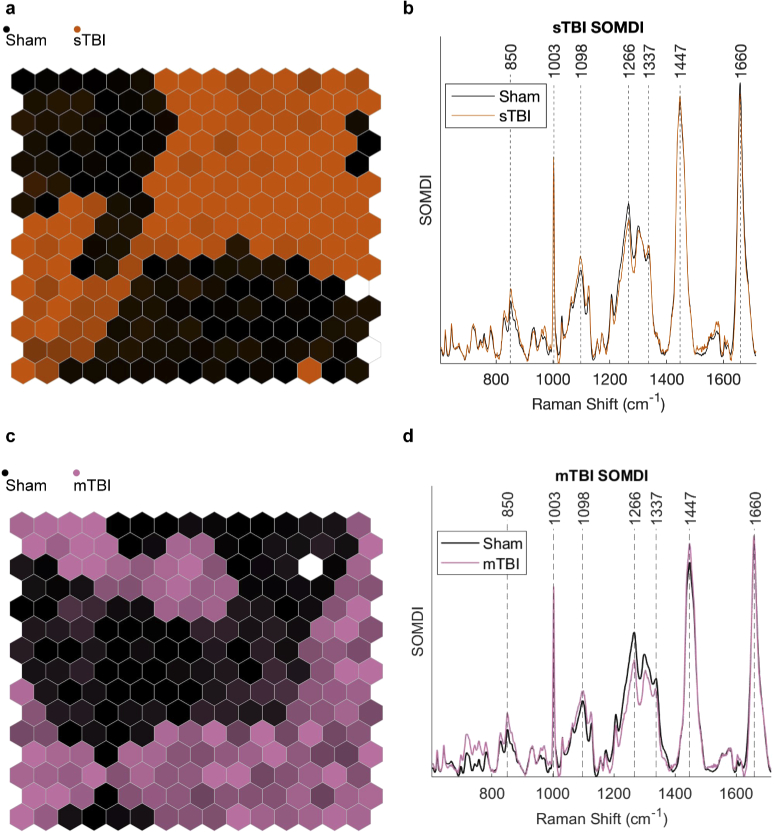
**a**, Clustering of Raman spectra from the retina for sTBI (orange) and sham (black) groups using a SOM. **b**, Features extracted (SOMDI) from SOM shown in (**a**), highlighting the Raman bands most influential to neurons in the SOM for sham and sTBI groups. **c**, Clustering of mTBI (purple) and sham (black) Raman spectra from the retina using a SOM. **d**, Features extracted from SOM in **c**, highlighting Raman bands for sham and mTBI groups.

Briefly, a SOM is a type of artificial neural network that is typically visualized as a 2D array of hexagonal neurons, which loosely tries to mimic the visual cortex in the brain; with neighboring neurons activating on similar inputs. The training process is performed iteratively by presenting an individual spectrum (ξ) from the training data, and finding the neuron that has previously activated on data most similar to the input, ξ. The winning neuron is then updated to become more likely to activate on data like ξ, along with neighboring neurons (but to a lesser degree). The result is neurons that are grouped according to particular features, as see in Fig. 2(a). Each neuron (hexagon) in the SOM is colored according to the type of data it activates from the training data (sham, mTBI or sTBI), providing immediate visualization of how the data is organized in groups.

A clear separation between sham and sTBI groups can be seen in the SOM shown in Fig. 2(a). To indicate neurons that activate on more than one injury state, color mixing is used according to the relative proportion of hits from each state. Using the SOMDI [15], it is possible to identify features in the Raman spectrum responsible for the clustering observed in the SOM. For sTBI: increases to the bands around 850, 1098 and 1337 ${\mathrm{cm}}^{-1}$, coupled to decreases in the bands around 1003, 1266 and 1660 ${\mathrm{cm}}^{-1}$ are observed, relative to the sham group (Fig. 2(b)). In comparison to sTBI, mTBI shows a poorer separation in the SOM (Fig. 2(c)), with a greater proportion of neurons activating on a mixture of mTBI and sham groups, particularly for neurons associated with mTBI. This is seen by mixing of the colors for injury states in the SOM. However, distinct regions are still present for both mTBI and sham groups. The same is true for the SOMDI of mTBI *vs* sham (Fig. 2(d)), with very few spectral regions of similarity between the two groups, indicating a greater degree of heterogeneity. Despite the increased variation, increases to the bands at 850, 1098 coupled to a decrease in the band at 1266 ${\mathrm{cm}}^{-1}$ are still observed for mTBI.

Following separation of the data from injured and healthy tissue, SKiNET can be used as a classifier. The classification method works by inputting a test sample into the trained network, and identifying which neuron is activated. The SOMDI associated with the activated neuron provides class data (based on the training inputs), which is used to make a prediction for the new sample. Models were optimized by using 10-fold cross validation on the training data to tune: the number of neurons in the SOM; initial learning rate; and training steps. Following optimization, the trained network was used to predict the previously unused test data, and showed good sensitivity for sTBI (82.0 ± 1.4 %). A poorer classification accuracy was obtained for sham (69.4 ± 0.9 %) and mTBI groups (75.1 ± 0.9 %), with a large proportion of sham data incorrectly classified as mTBI and *vice versa* (Table 5). Similar results were obtained by splitting the data according to ipsilateral (side of injury) and contralateral retinae (Table S1), and in comparison to the cross validation accuracy against the training data (Table S2).

**Table 5.** Summary of classification accuracy as a confusion matrix for: sham, mTBI and sTBI groups using trained SKiNET against test data. Data shown is the average classification accuracy across 10 SOM initializations, trained using Raman spectra from flat mounted mouse retina (both eyes).

		Predicted		
		**Sham (%)**	**mTBI (%)**	**sTBI (%)**

Actual	**Sham (%)**	**69.4**	24.0	6.6
	**mTBI (%)**	17.9	**75.1**	6.9
	**sTBI (%)**	7.9	10.0	**82.0**

### 3.2 Corresponding changes to brain spectra

In contrast to the retina, Raman spectra of brain tissue from the site of injury showed more dramatic and consistent changes across injury groups. Even in the average spectra over all samples (Fig. 1(d)), there are visible differences to the bands around 1266 ${\mathrm{cm}}^{-1}$ and 1660 ${\mathrm{cm}}^{-1}$. This observation is supported by SKiNET analysis applied to the spectra from brain tissue in the contusion core, using the same methodology described in the previous section. Spectra from the test data were classified with an accuracy of (100 ± 0.0 % ) for sham, (94.5 ± 0.9 % ) for mTBI and (91.2 ± 1.3 % ) for sTBI. Furthermore, the extracted features using the SOMDI (Fig. S4) closely resemble the average spectra.

**Fig. 3.**
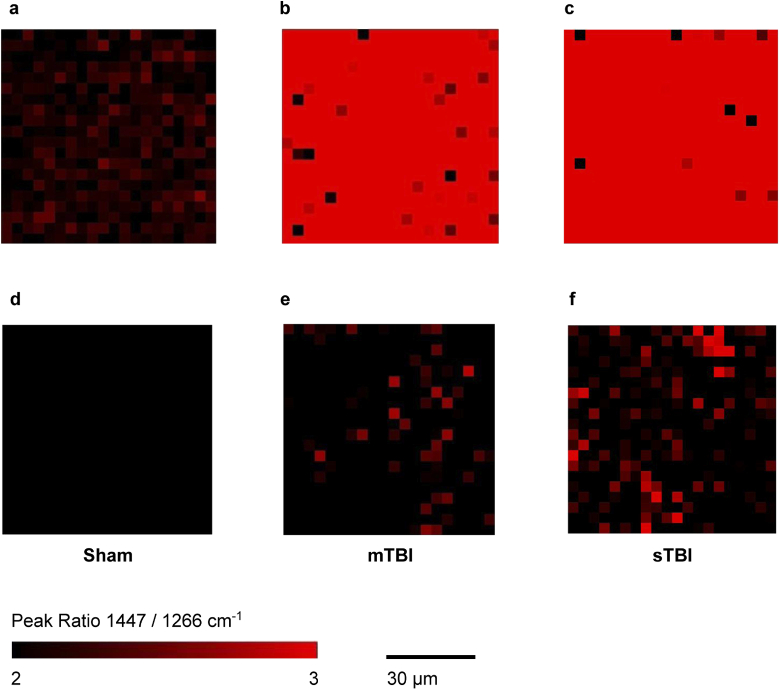
False colored Raman maps showing the peak ratio at 1447 / 1266 ${\mathrm{cm}}^{-1}$ for a single brain tissue sample from Sham (**a**), mTBI (**b**), and STBI (**c**) groups. **d-f**, Corresponding Raman maps of ipsilateral tissue of retina from the same mice. Raman maps for all samples are shown in Fig. S5-S6.

**Fig. 4.**
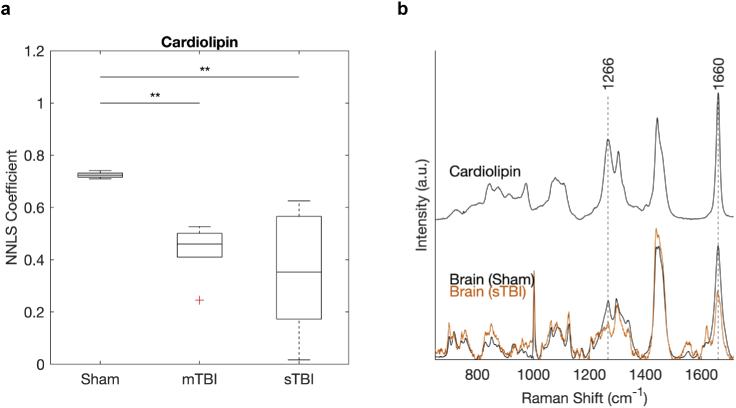
**a**, Change in the relative contribution from cardiolipin for sham, mTBI and sTBI. Boxplot shows the NNLS coefficient fitted to the average map spectrum measured from each brain sample at the injury site, in the range 1200-1714 ${\mathrm{cm}}^{-1}$. A statistically significant difference determined by one-way ANOVA exists in cardiolipin for mTBI (p = 0.0090) and sTBI (p = 0.0011) compared to the sham. There is no statistically significant difference between mTBI and sTBI (*p < 0.05, **p < 0.01). **b**, Spectra for cardiolipin (using data from Krafft *et al.* [19]) and average spectra for brain tissue from sham and sTBI.

A decrease to the band at 1266 ${\mathrm{cm}}^{-1}$ in TBI samples appears to be a key feature from the analysis of both brain and retina. Visually, this can be observed using false colored Raman maps of the ratio of the band at 1447 vs 1266 ${\mathrm{cm}}^{-1}$ (Fig. 3). The band at 1266 ${\mathrm{cm}}^{-1}$ is assigned to CH bending modes, and commonly associated with amide groups in lipids and proteins [20]. Since the brain contains nearly 60 % fat and the Raman signature for all 12 major and minor brain specific lipids have been well characterized [19,22], we attempted to decompose the changes due to the lipid contribution. NNLS fitting was performed against average spectra from brain tissue from each sample (Fig. S2d-f), using a library of component spectra, which included the raw data for brain specific lipids, cardiolipin (provided by Krafft *et al.* [19]) and cytochrome c. Fitting was performed in the range 1200-1714 ${\mathrm{cm}}^{-1}$, to identify the relative contributions in each tissue sample for sham, mTBI and sTBI. The resultant fitting coefficients for each lipid spectrum are therefore proportional to the lipid concentration measured within each tissue sample. A one-way ANOVA shows a statistically significant difference in the contribution (compared to the sham) from cardiolipin (Fig. 4(a)), linked to the decrease in the bands at 1266 and 1660 ${\mathrm{cm}}^{-1}$ (Fig. 4(b)) in TBI for both moderate and severe TBI *vs* the sham. No significant change was observed between injury severity for cardiolipin. A small decrease in the fitting coefficient for cholesterol was observed, along with an increase in sphingomyelin, but were not statistically significant (Table S3-S5).

## 4. Discussion

Through the use of SKiNET, subtle changes to spectral features have been identified for TBI, further highlighting the value of SOMs in the analysis of Raman spectra. The observed change to the band 1266 ${\mathrm{cm}}^{-1}$ has been shown to be present for both the brain and retina, proportional to injury severity (Fig. 3). However, whilst Raman maps of brain tissue show a strong and uniform relative decrease at 1266 ${\mathrm{cm}}^{-1}$, tissue from the retina appears more heterogeneous. This suggests that future applications of the technology may require targeted scanning over specific areas of the retina (e.g. optic disc), and identify a number of spectra above a certain threshold for TBI. Furthermore, the change seen in Fig. 3 was not observed consistently across all samples, especially from contralateral tissue (Fig. S6). One implication is that a change to a single Raman band in isolation is not enough to classify tissue, emphasizing the importance of tools such as SKiNET, which use all of the available data.

From brain tissue, obvious and consistent changes were observed to spectra in response to injury, which is in contrast to the subtle changes seen in the previous work by Surmacki *et al.* [8]. An explanation for this discrepancy is that we collected a large number of spectra over an area for each sample, whilst following the surface topography. Hemorrhaging that was clearly visible in the contusion core from mTBI and sTBI samples of the brain (Fig. 1(a)) led to a strong background autofluorescence, which we have attributed to the near infrared absorption of deoxyhemoglobin [23]. Whilst this did not effect measurements from the retina, this may have led to artifacts in the baseline correction, such as the small bands observed between 1447 and 1660 ${\mathrm{cm}}^{-1}$.

### 4.1 Biochemical attribution

Attribution and deconvolution of Raman spectra to biological species from spectra of tissue remains challenging, which we have attempted through the use of NNLS fitting. The choice of fitting library followed previous qualitative analysis [8], and included cardiolipin and cyctochrome c as markers of mitochondrial activity. Cardiolipin is found exclusively in the inner mitochondrial membrane, playing a crucial role in cell metabolism and signaling; including apoptosis. A decrease in cardiolipin concentration following cortical impact has recently been shown using mass spectrometry, where both the importance in relation to TBI and opportunity for future therapeutics were highlighted [16]. Furthermore, the release of cardiolipin microparticles following TBI induced cell damage has been show to compromise the blood brain barrier, and so plays a major role in the resulting biochemical cascade and metabolic disruption [24]. These findings are also consistent with earlier studies using Raman spectroscopy to assess TBI in mice, where the authors concluded a link between the observed spectral changes and apoptosis *via* comparison to immunohistochemistry of the samples [25]. Cytochrome c is a protein found in the inner membrane of mitochondria, where a complementary change to cardiolipin was expected. Small coefficients were fitted for cytochrome c in the mTBI and sTBI groups, which were not present in the sham group. This adds additional weight to the conclusion that the observed changes in response to injury are a result of metabolic distress. Although these results are encouraging, it should be noted that the spectral bands associated with cardiolipin are also present in several proteins, and so these changes cannot solely be attributed to cardiolipin [26].

### 4.2 Translation and future work

Despite the obvious challenges for Raman spectroscopy of the eye, it appears to be an area of active development, with Stiebing *et al.* [13] recently showing how spectra can be safely collected from *ex-vivo* human retina using a synthetic model of the optical parameters of the eye, in place of a microscope objective. The spectra acquired through such a low numerical aperture geometry are inevitably noisy, or require long acquisition times. In the present study, the important issue of eye safety limits have not been addressed, and a traditional Raman microscope geometry used. However, short acquisition times meant that individual spectra used as training inputs were noisy and yet, *via* SKiNET these were used to accurately identify TBI severity. Now that we have established a preliminary proof of concept, a study using a larger animal model of TBI (e.g. porcine) could be used demonstrate *in-vivo* viability.

## 5. Conclusions

For the first time, we have shown that Raman spectroscopy can be used to effectively and accurately identify TBI from tissue samples of the retina, coupled to chemical changes from a cortical impact to the brain. Machine learning using the SOMDI and SKiNET has been used to extract the subtle spectral changes present from the retina, and shown these to be in line with the measured changes to brain tissue. Consistent changes were observed in particular for the band at 1266 ${\mathrm{cm}}^{-1}$ both in brain and retina tissue for mTBI and sTBI, when compared to the sham group. Raman spectroscopy represents a unique opportunity for TBI monitoring throughout the patient journey from pre-hospital assessment, to intensive care and follow up examinations. In demonstrating a fundamental ability to study chemical changes from eye tissue as a result of TBI, we begin to push the boundaries of Raman spectroscopy of the eye beyond purely ophthalmic applications; opening a new window to study neurological changes.

## Notes

See Supplement 1 for supporting content.
