# Supplementary material for: Spectroscopic detection of traumatic brain injury severity and biochemistry from the retina
Source: Biomed Opt Express. 2020 Oct 8;11(11):6249–61. doi: 10.1364/BOE.399473 (PMC7687955; doi:10.1364/BOE.399473)
Supplement: Supplementary file 1 [file boe-11-11-6249-s001.pdf]

Supplemental Document

Biomedical Optics EXPRESS

Spectroscopic detection of traumatic brain injury severity and biochemistry from the retina: supplement

CARL BANBURY,[1] IAIN STYLES,[2] NEIL EISENSTEIN,[1] ELISA R. ZANIER,[3] GLORIA VEGLIANTE,[3] ANTONIO BELLI,[4] ANN LOGAN,[4] AND POLA GOLDBERG OPPENHEIMER[1,*]

[1] *School of Chemical Engineering, The University of Birmingham, Edgbaston, Birmingham, B15 2TT, UK*
[2] *Computer Science, The University of Birmingham, Edgbaston, Birmingham, B15 2TT, UK*
[3] *Department of Neuroscience, Istituto di Ricerche Farmacologiche Mario Negri IRCCS, Milan, Italy*
[4] *Institute of Inflammation and Ageing, The University of Birmingham, Edgbaston, Birmingham, B15 2TT, UK*
[*] *P.GoldbergOppenheimer@bham.ac.uk*
https://anmsa.com

This supplement published with The Optical Society on 8 October 2020 by The Authors under the terms of the Creative Commons Attribution 4.0 License in the format provided by the authors and unedited. Further distribution of this work must maintain attribution to the author(s) and the published article's title, journal citation, and DOI.

Supplement DOI: https://doi.org/10.6084/m9.figshare.12991118

Parent Article DOI: https://doi.org/10.1364/BOE.399473

Supplementary Material

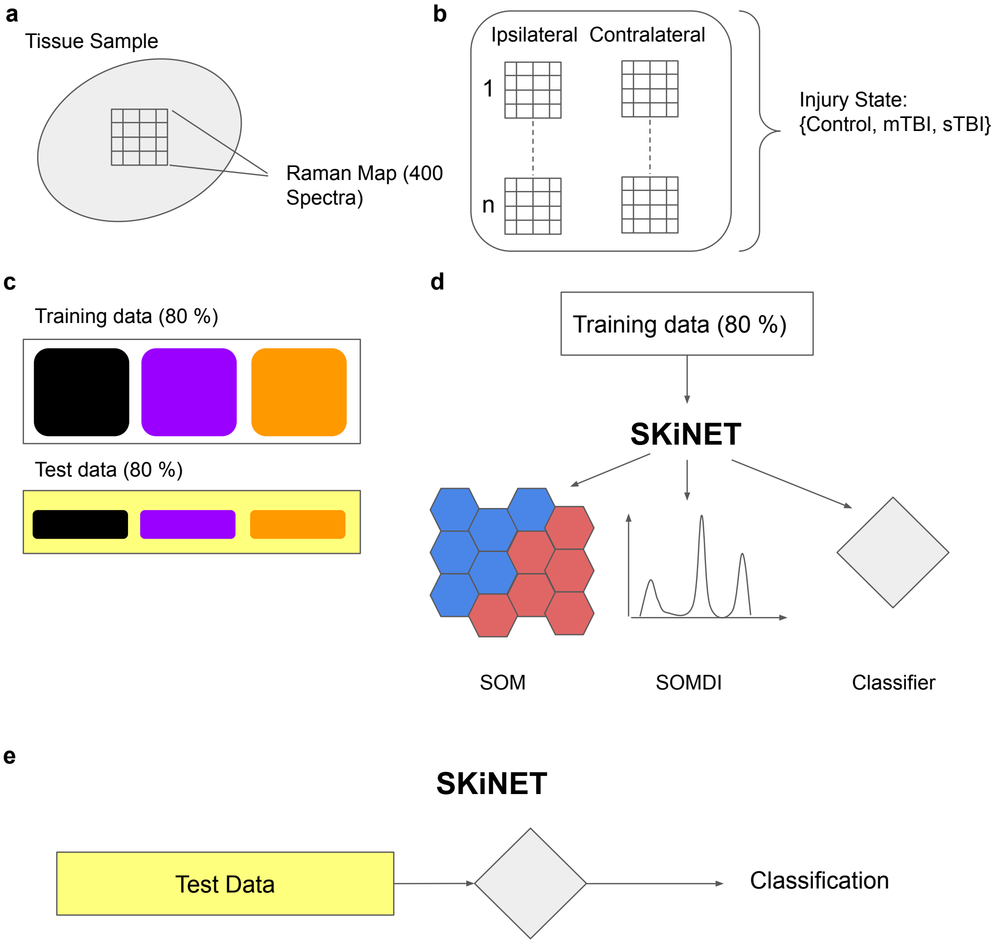

Fig. S1. Illustration of data analysis workflow for retina tissue using SKiNET. Spectra measured from Raman maps (**a**) of flat mounted retina (n=6) are grouped according to injury state (**b**). A 20 % partition of the data is randomly selected and reserved as test data (**c**). The remaining 80 % is input into SKiNET, which directly provides dimensionality reduction (SOM), feature extraction (SOMDI) and classification **d**. SKiNET is optimized on the training data using cross validation, and adjusting the available parameters (number of neurons, initial learning rate and number of training steps) to maximize the classification accuracy on the training data. Finally, the optimized model is shown the previously unused test data and asked to classifify each spectrum as either sham, mTBI or sTBI (**e**).

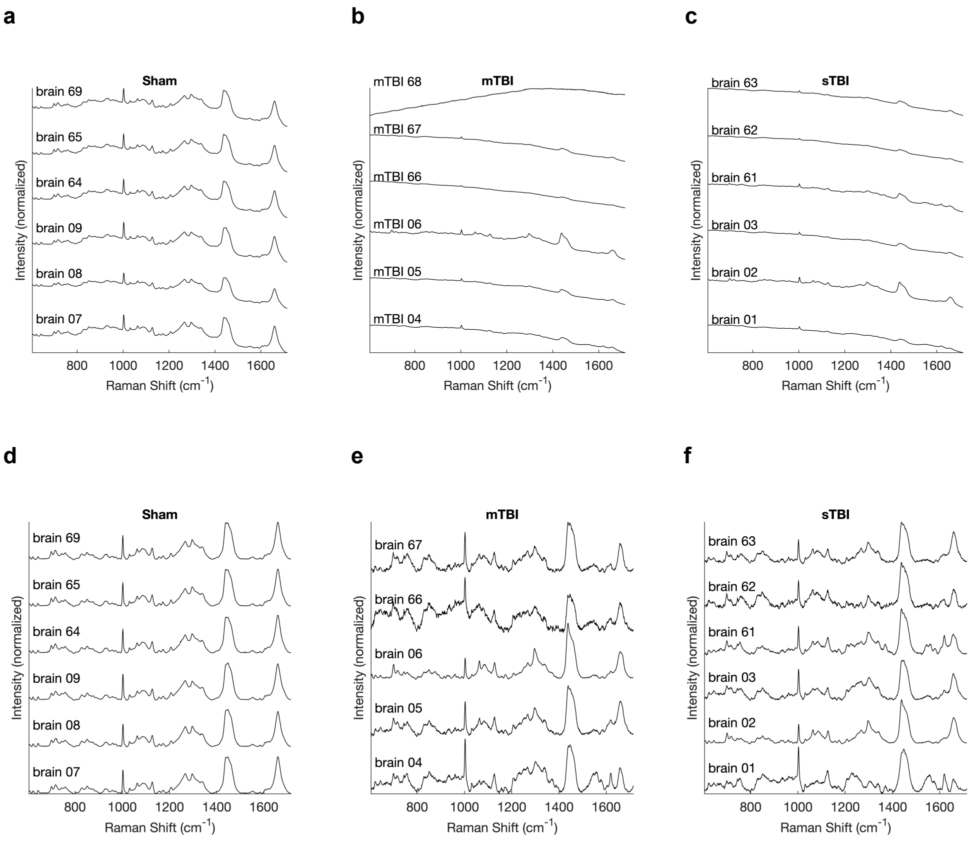

Fig. S2. Average Raman spectra collected from the injury site on left hemisphere of brain (n=6) for mTBI (**a**), sTBI (**b**), and sham (**c**) groups. Data for each tissue sample were collected as a Raman surface map (400 points) following the surface topography. **d-f**, Average Raman spectra for sham, mTBI and sTBI samples following baseline subtraction using an intelligent spline fit.

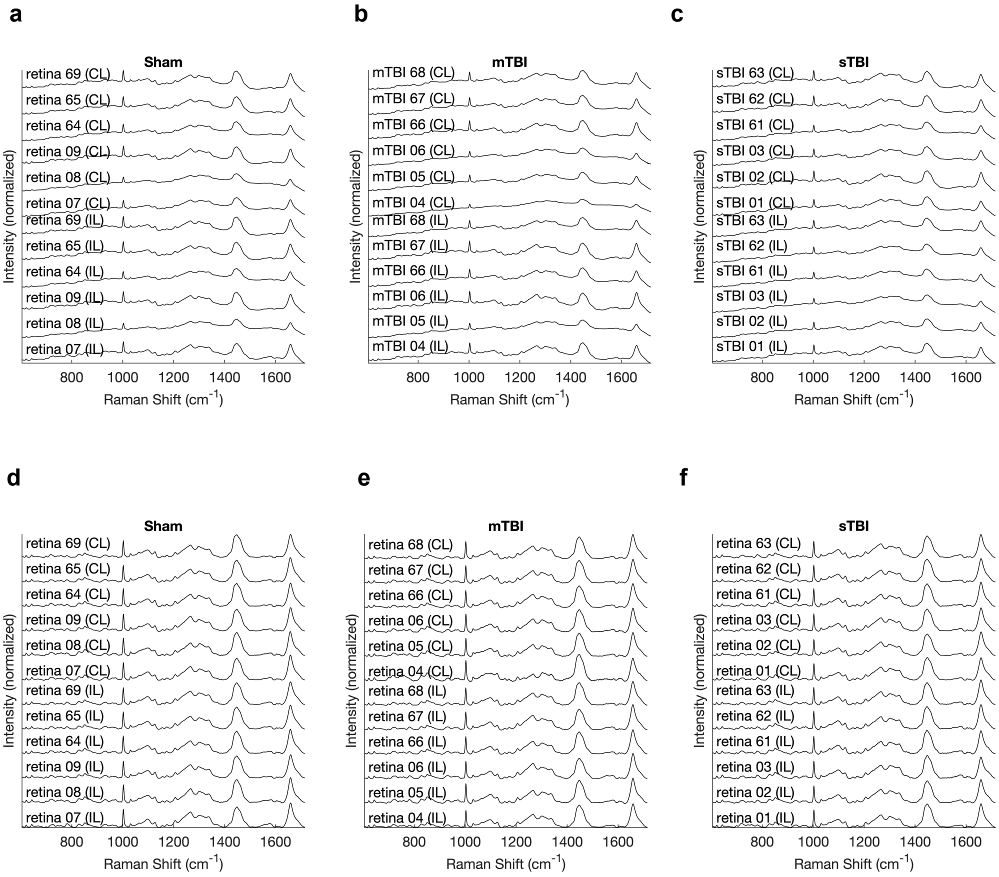

Fig. S3. Average Raman spectra collected from ipsilateral (IL) and contralateral (CL) flat mounted retina (n=6) for mTBI (**a**), sTBI (**b**), and sham (**c**) groups. Data for each tissue sample were collected as a Raman surface map (400 points) following the surface topography. **d-f**, Average Raman spectra for sham, mTBI and sTBI samples following baseline subtraction using an intelligent spline fit.

Model	Sham (%)	mTBI (%)	sTBI (%)
Bilateral	69.4 (± 0.9)	75.1 (± 0.9)	82.0 (± 1.4)
Ipsilateral	81.5 (± 0.8)	76.7 (± 1.9)	86.2 (± 1.4)
Contralateral	76.6 (± 1.6)	76.6 (± 1.2)	88.7 (± 0.9)

Table S1. Classification accuracy of TBI using Raman spectra of retina, modeled using data from both eyes (bilateral), eyes from the side of injury only (ipsilateral) and contralateral eyes. Results show the average over 10 SOM initializations and standard deviation in brackets.

		Predicted		
		**Sham (%)**	**mTBI (%)**	**sTBI (%)**
Actual	**Sham (%)**	**67.8**	24.9	7.3
	**mTBI (%)**	17.6	**76.1**	8.6
	**sTBI (%)**	8.8	13.6	**77.7**

Table S2. Summary of cross-validation accuracy as a confusion matrix for: sham, mTBI and sTBI from flat mounted mouse retina (both eyes) on 80 % partition of training data. Model trained using 400 neurons, with an initial learning rate of 0.2, and 57600 training steps.

Lipid	#07	#08	#09	#64	#65	#69	Average
Cardiolipin	0.74	0.71	0.72	0.73	0.71	0.72	0.72
Cholesteryl ester	0	0	0	0	0	0	0
Cholesterol	0.17	0.19	0.21	0.16	0.17	0.16	0.18
Cytochrome C	0	0	0	0	0	0	0
Galactocerebroside	0	0	0	0	0	0	0
Ganglioside	0	0	0	0	0	0	0
Sphyingomyelin	0.07	0.08	0.04	0.09	0.10	0.10	0.08
Phosphatidylcholine	0	0	0	0	0	0	0
Phosphatidylserine	0	0	0	0	0	0	0
Phosphatidylinositol	0	0	0	0	0	0	0
Phosphatidylethanolamine	0	0	0	0	0	0	0
Sulfatide	0	0	0	0	0	0	0
Triacylglyceride	0	0.01	0.01	0	0	0	0
R^2	0.88	0.89	0.87	0.86	0.86	0.86	0.87

Table S3. Decomposition of contribution from brain lipids (rows) in average Raman spectra of brain samples (columns) from contusion core for sham group using NNLS fitting. Each sample was fitted against the set of component spectra in the range 1200 - 1714 cm^{-1}.

Lipid	#04	#05	#06	#66	#67	Average
**Cardiolipin**	0.24	0.53	0.41	0.49	0.50	0.43
Cholesteryl ester	0	0	0	0	0	0
**Cholesterol**	0.17	0.16	0.20	0.08	0.19	0.16
Cytochrome C	0.35	0.10	0	0.25	0.02	0.14
Galactocerebroside	0.14	0	0	0.15	0	0.06
Ganglioside	0	0	0	0	0	0
**Sphyingomyelin**	0	0.16	0.39	0	0.14	0.14
Phosphatidylcholine	0.13	0	0.05	0	0.10	0.06
Phosphatidylserine	0.016	0	0	0	0	0
Phosphatidylinositol	0	0	0	0	0	0
Phosphatidylethanolamine	0	0	0	0	0	0
Sulfatide	0	0	0	0	0	0
**Triacylglyceride**	0	0.04	0	0	0.08	0.03
R^2	0.8	0.84	0.92	0.75	0.89	0.84

Table S4. Decomposition of contribution from brain lipids (rows) in average Raman spectra of brain samples (columns) from contusion core for mTBI group using NNLS fitting. Lipids that have non-zero fitting coefficients in the sham group are highlighted in bold. Each sample was fitted against the set of component spectra in the range 1200 - 1714 cm^{-1}.

Lipid	#01	#02	#03	#61	#62	#63	Average
**Cardiolipin**	0.17	0.51	0.57	0.19	0.02	0.63	0.35
Cholesteryl ester	0	0	0	0	0	0	0
**Cholesterol**	0.18	0.19	0.22	0.16	0.13	0.1	0.16
Cytochrome C	0.41	0	0.07	0.22	0	0	0.12
Galactocerebroside	0	0	0	0	0	0	0
Ganglioside	0	0	0	0	0	0	0
**Sphyingomyelin**	0	0.32	0.1	0.18	0.12	0.25	0.16
Phosphatidylcholine	0.02	0	0	0.22	0.57	0	0.13
Phosphatidylserine	0.01	0	0	0	0	0	0
Phosphatidylinositol	0	0	0	0	0	0	0
Phosphatidylethanolamine	0	0	0	0	0	0	0
Sulfatide	0	0	0	0	0	0	0
**Triacylglyceride**	0.27	0.03	0.03	0.04	0.29	0.06	0.12
R^2	0.62	0.90	0.86	0.84	0.77	0.84	0.81

Table S5. Decomposition of contribution from brain lipids in average Raman spectra of brain samples from contusion core for sTBI group using NNLS fitting. Lipids that have non-zero fitting coefficients in the sham group are highlighted in bold. Each sample was fitted against the set of component spectra in the range 1200 - 1714 cm^{-1}.

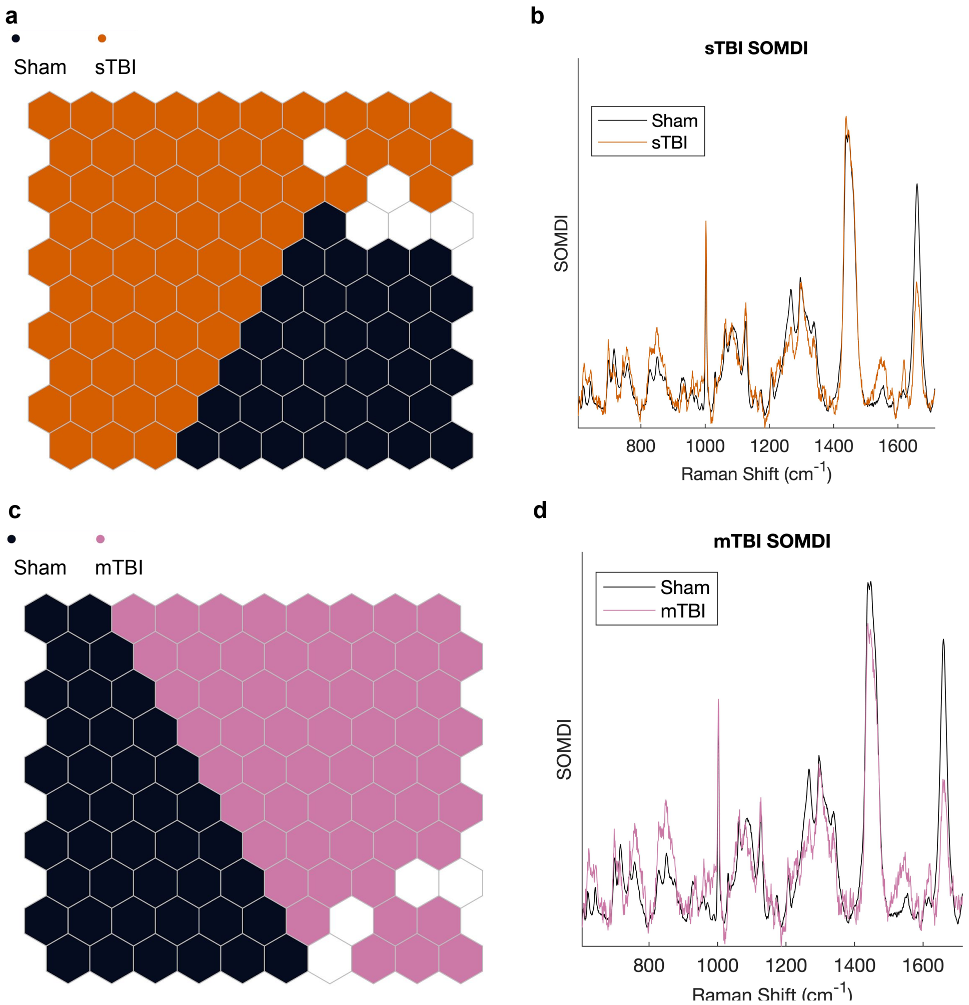

Fig. S4. **a**, Clustering of Raman spectra from the brain in the contusion core for sTBI (orange) and sham (black) groups using a SOM. **b**, Features extracted (SOMDI) from SOM shown in (**a**), highlighting the Raman bands most influential to neurons in the SOM for sham and sTBI groups. **c**, Clustering of mTBI (purple) and sham (black) Raman spectra from the brain in the contusion core using a SOM. **d**, Features extracted from SOM in **c**, highlighting Raman bands for sham and mTBI groups.

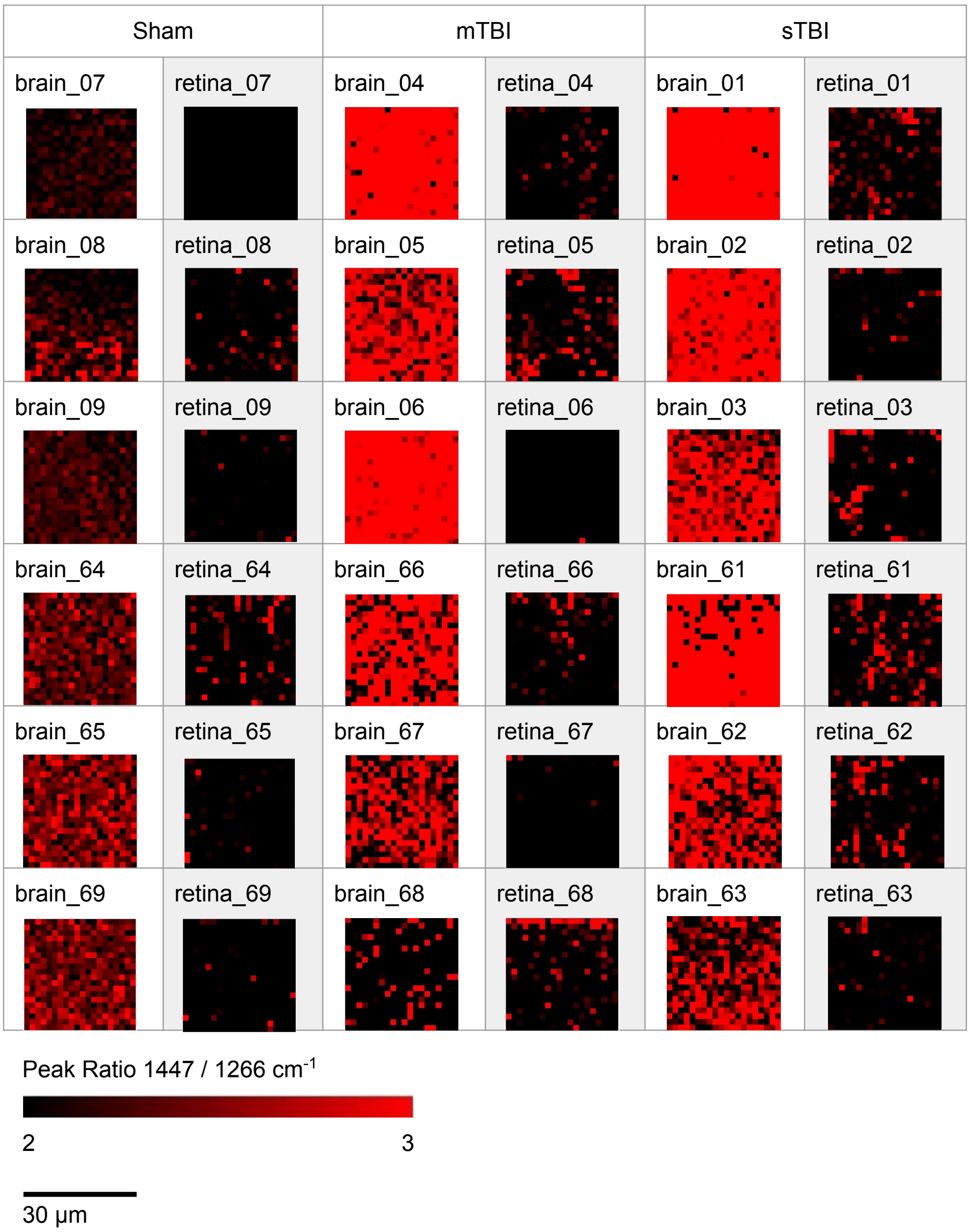

Fig. S5. False colored Raman maps for whole brain tissue from the contusion core, and for corresponding ipsilateral flat mounted retina samples. Maps are colored according to the ratio between the bands at 1447 and 1266 cm^{-1}.

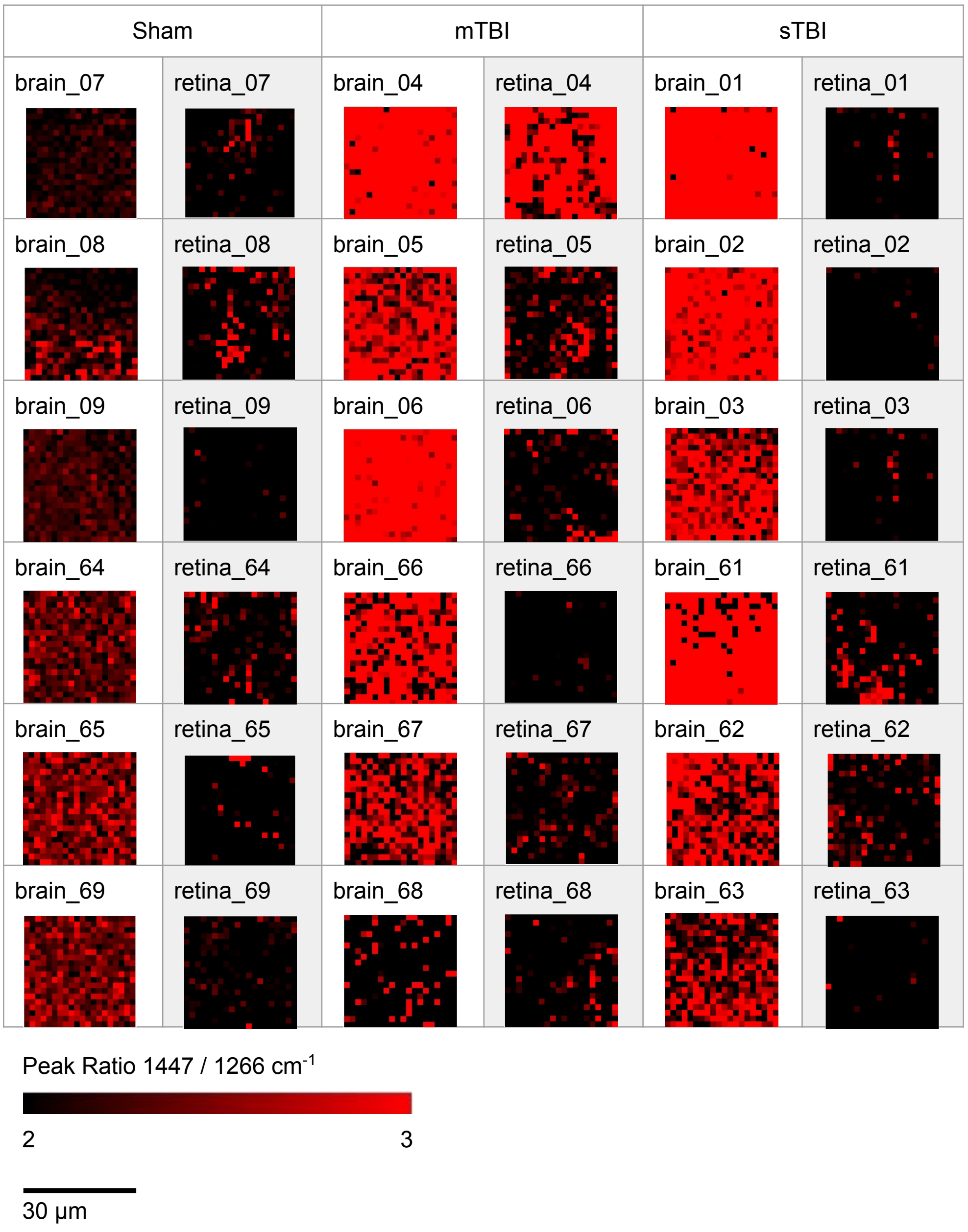

Fig. S6. False colored Raman maps for whole brain tissue from the contusion core, and for corresponding contralateral flat mounted retina samples. Maps are colored according to the ratio between the bands at 1447 and 1266 cm^{-1}.
